# Correlation of cytomegalovirus viral load between whole blood and plasma of congenital cytomegalovirus infection under valganciclovir treatment

**DOI:** 10.1186/s12879-023-07995-6

**Published:** 2023-01-19

**Authors:** Yuka Torii, Ichiro Morioka, Yasumasa Kakei, Kazumichi Fujioka, Yu Kakimoto, Naoto Takahashi, Tetsushi Yoshikawa, Hiroyuki Moriuchi, Akira Oka, Yoshinori Ito

**Affiliations:** 1 Department of Pediatrics, Nagoya University Graduate School of Medicine, Nagoya, 466-8550 Japan; 2 Department of Pediatrics and Child Health, Nihon University School of Medicine, 30-1 Oyaguchi, Kami-cho, Itabashi-ku, Tokyo, 173-8610 Japan; 3 Clinical and Translational Research Center, Kobe University Hospital, Kobe, 650-0017 Japan; 4 Department of Pediatrics, Kobe University Graduate School of Medicine, Kobe, 650-0017 Japan; 5 Department of Pediatrics, The University of Tokyo, Tokyo, 113-8655 Japan; 6 Department of Pediatrics, Fujita Health University School of Medicine, Toyoake, 470-1192 Japan; 7 Department of Pediatrics, Nagasaki University Graduate School of Biomedical Sciences, Nagasaki, 852-8501 Japan; 8 Saitama Prefectural Children’s Medical Center, Saitama, 330-8777 Japan

**Keywords:** Congenital CMV infection, PCR, Plasma, Valganciclovir, Whole blood

## Abstract

**Background:**

Congenital cytomegalovirus (CMV) infection (cCMV) can cause sensorineural hearing loss and neurodevelopmental disabilities in children. Oral valganciclovir (VGCV) therapy has been reported to improve long-term audiological and neurodevelopmental outcomes in patients with cCMV. The levels of CMV DNA in whole blood have been monitored in previous studies. However, quantitative methods using whole blood have not been standardized. Recently, the plasma viral load has been standardized and widely used in CMV-associated diseases.

**Methods:**

CMV viral loads in whole blood and plasma were serially measured in 24 patients with a confirmatory diagnosis of cCMV during oral VGCV therapy using an in-house real-time PCR assay. Plasma samples were assayed using the Cobas 6800 system (Roche Diagnostics) in addition to an in-house assay.

**Results:**

Plasma CMV viral loads were remarkably decreased at the end of therapy compared to before therapy. A significant correlation of CMV levels between whole blood and plasma was observed (Spearman’s ρ = 0.566). The levels of CMV DNA before therapy were significantly correlated with the period of decreasing the viral loads to below the detection limit, not only in whole blood (Spearman’s ρ = 0.901) but also in plasma (Spearman, ρ = 0.804). Finally, CMV viral loads between the in-house assay and commercially available standardized assay in 75 plasma samples with positive PCR results for CMV were compared; a significant correlation was observed between the results of both assays.

**Conclusions:**

There was a significant correlation between the two assays (Spearman, ρ = 0.882), suggesting that CMV plasma viral loads measured by the standardized assay are widely used to monitor the levels of CMV DNA in patients with cCMV during oral VGCV therapy.

## Background

Cytomegalovirus (CMV) is one of the most common pathogens associated with mother-to-child infections. Most congenital CMV (cCMV) infants are born without any symptoms (asymptomatic), whereas 10–15% of cCMV infants show any physical symptoms (symptomatic) [1]. The clinical manifestations of cCMV infection include small gestational age, microcephaly, petechiae, jaundice, hepatosplenomegaly, and purpura. Intracranial calcification, periventricular cysts or ventriculomegaly, sensorineural hearing loss (SNHL), and retinitis can be detected by examination. Developmental delays or other neurological symptoms, such as epilepsy, can appear as the patient matures. SNHL can also become apparent as a relatively late symptom. An epidemiological study revealed a cCMV prevalence of 3.3 in 1000 births in Japan [2]. Polymerase chain reaction (PCR) assays have been used to diagnose cCMV and have become the standard diagnostic method [3]. In Japan, qualitative nucleic acid amplification tests for diagnosing cCMV were approved by government health insurance in 2018, resulting in an increase in the number of patients with a confirmatory diagnosis of cCMV.

Since Kimberlin et al. showed that oral valganciclovir (VGCV) treatment for 6 months improved hearing and neurodevelopmental outcomes in infants with cCMV [4], the efficacy and safety of VGCV therapy have been recognized in several studies [5–8]. However, VGCV therapy for symptomatic infants with cCMV is not the standard therapy in Japan. Therefore, the Japanese congenital cytomegalovirus study group conducted a multicenter, open-label, single-arm clinical trial [9, 10]. To evaluate the viral load during VGCV treatment, the amount of CMV DNA in whole blood has been measured using quantitative PCR assays in previous studies. Plasma is widely used for measuring CMV viral load in other diseases under immunocompromised conditions, and a standardized method has been commercially available [11]. The correlation of CMV levels between whole blood and plasma has been analyzed in several studies in immunocompromised patients treated with antivirals, such as donors of hematopoietic stem cell transplantation (HSCT) and organ transplantations [12–15]. However, the correlation between CMV loads in whole blood and plasma has not yet been investigated in cCMV patients. This study aimed to serially measure CMV viral loads in whole blood and plasma in patients with cCMV infection during oral VGCV therapy using an in-house real-time PCR assay. The results from our in-house assay and commercially available standardized assay were compared. If CMV viral loads in plasma are practicable likewise viral loads in whole blood, the standardized commercial quantitative PCR assay can be applied to monitor CMV viral loads in patients undergoing antiviral therapy.

## Methods

### Patients and sample collection

To evaluate CMV viral loads, whole blood, plasma, and urine samples were obtained from patients with cCMV in a non-randomized, prospective, open-label, multicenter clinical trial conducted by a Japanese cCMV study group from February to October 2020. The study protocol for this clinical trial has been previously detailed [9]. Briefly, a patient was included in the study if they met all the following five criteria: (1) confirmation of positive CMV-DNA in urine by an in vitro diagnostic test within 21 days of age; (2) any neurological symptoms for congenital CMV, such as microcephaly, hydrocephalus or periventricular enlargement, ventricular calcification, cerebral cortical hypoplasia or white matter injury, retinal chorioretinitis, or abnormal auditory brainstem response (ABR); (3) informed consent obtained within 60 days of age; (4) gestational age > 32 weeks at birth; and (5) body weight > 1800 g at study enrollment. Blood and urine samples were obtained at every hospital visit during viral VGCV therapy (before therapy, every week until the 6th week of therapy, and every month from the 2nd to 6th month of therapy). After the screening for patient registration in this study, the participants were orally administered 16 mg/kg VGCV twice daily for 6 months during the treatment period. The CMV viral load was evaluated in whole blood and urine immediately after sample collection. Plasma samples were stored at − 80 °C until retrospectively analyzed.

Plasma samples from patients with positive PCR results for whole blood samples based on the in-house assay were used to compare the two real-time PCR assay systems for CMV detection. These patients included those with cCMV (n = 20), HSCT recipients (n = 30), and liver transplantation recipients (n = 25), and this patient group was named the “plasma validation group”.

### Real-time PCR assay for CMV

For the in-house assay, DNA was extracted from 200 µL of whole blood or plasma using a QIAamp DNA blood mini kit (Qiagen, Hilden, Germany) and from 140 µL of urine using a QIAamp Viral RNA Mini Kit (Qiagen). Viral loads were measured via real-time PCR using QuantStudio 3 (Applied Biosystems, Foster City, CA, USA) in a total volume of 25 µL composed of 5 µL DNA, 12.5 µL Taqman Fast Advanced Mix (Applied Biosystems), 0.05 µL each of 50 µM sense and antisense primers, 0.025 µL of 100 µM probe, and 7.125 µL of nuclease-free water [16]. All assays were performed in triplicate. Plasma samples of the validation group were assayed using both an in-house assay and the Cobas 6800 system (Roche Diagnostics, Indianapolis, IN, USA) [17] in addition to an in-house assay. DNA was extracted from 500 µL of plasma using a fully automated Cobas 6800 system.

The lower limit of detection of the in-house assay was 250 IU/mL (throughout the assay including the DNA extraction and PCR reaction). The lower limit of detection of the Cobas 6800 system was 34.5 IU/mL (PCR reaction).

### Statistical analysis

Viral load between whole blood and plasma and the two assay systems (in-house assay vs. commercial assay) were compared using Spearman’s rank correlation coefficient. Statistical analyses were performed using the SPSS software ver. 28 (IBM, Armonk, NY, USA).

## Results

### CMV viral load in the patients with cCMV under oral VGCV therapy

A total of 25 infants were enrolled in this clinical trial. One patient was excluded because of severe epilepsy before initiating VGCV therapy. The therapy was administered to 21 infants but was discontinued for 3 infants during the therapy because of neutropenia. The effectiveness of VGCV has also been reported [10]. CMV viral loads in whole blood, plasma, and urine samples from baseline to the end of therapy were analyzed in 21 patients (276 sample collection points).

**Fig. 1:**
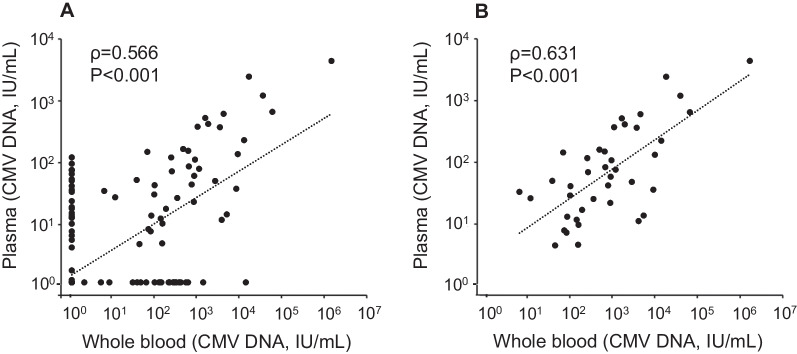
Correlation between the levels of cytomegalovirus (CMV) DNA in plasma and those in whole blood in congenital CMV (cCMV) patients. **A** All samples are plotted and analyzed (n = 276). **B** Samples in which CMV DNA was positive both in whole blood and in plasma are plotted and analyzed (n = 40). The levels of CMV DNA were measured using the in-house real-time PCR assay. Viral load was compared between whole blood and plasma using Spearman’s rank correlation coefficient

The CMV levels in whole blood and plasma samples were compared at each sample collection point in all patients (Fig. 1). A significant correlation was obtained, but an inconsistent qualitative result was observed in some samples; in these samples, CMV DNA was positive in whole blood but negative in plasma (whole blood+/plasma− pattern), or CMV DNA was positive in plasma but negative in whole blood (whole blood−/plasma+ pattern) (Fig. 1A). The differences in the levels of CMV DNA between whole blood and plasma appeared on more than one logarithmic scale in most samples.

**Fig. 2:**
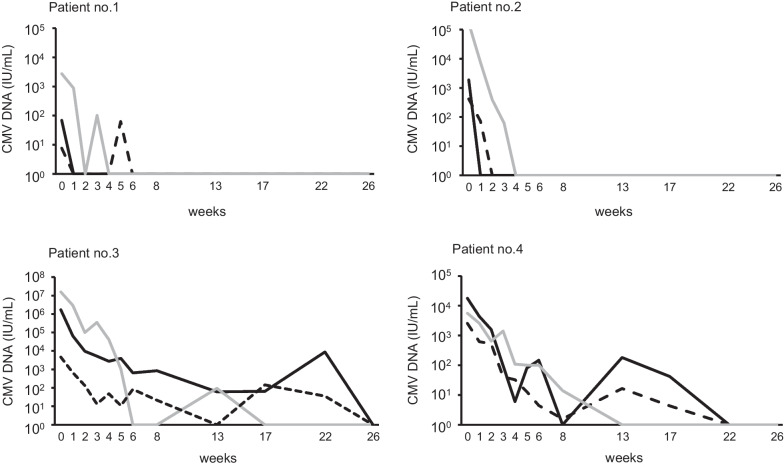
Results of monitoring CMV viral loads in representative four cases with cCMV under the oral valganciclovir (VGCV) therapy. The levels of CMV DNA were measured using the in-house real-time PCR assay. Black line, dynamics of CMV load in whole blood; dotted line, dynamics of CMV load in plasma; grey line, dynamics of CMV load in urine

**Fig. 3:**
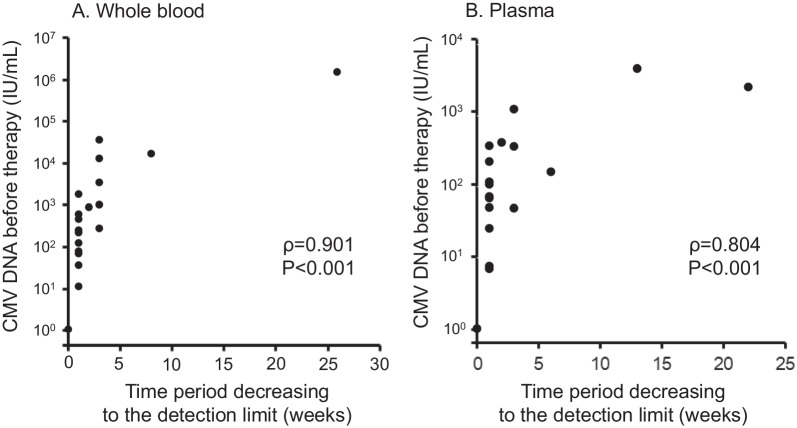
Correlation between the levels of CMV before the oral VGCV therapy and a period of decreasing to under detection limit in cCMV patients. **A** Results in whole blood (n = 21). **B** Results in plasma samples (n = 21). The levels of CMV DNA were measured using the in-house real-time PCR assay. The viral load, period and plasma were compared using Spearman’s rank correlation coefficient

Kinetics of CMV viral loads in whole blood, plasma, and urine are shown in the four representative cases shown in Fig. 2. In most cases, CMV viral loads fell below the detection limit within the first 6 weeks, as shown in patients 1 and 2. In contrast, the levels of CMV did not decrease in the 2 cases (patients 3 and 4). The CMV viral loads before therapy initiation were relatively high in these patients. Next, the levels of CMV DNA before therapy were well correlated with the period of the viral loads decreasing to below the detection limit, not only in whole blood (Spearman’s ρ = 0.901) but also in plasma (Spearman, ρ = 0.804) (Fig. 3).

There was no apparent difference in the CMV DNA clearance between whole blood and plasma. The CMV DNA in the two components decreased simultaneously, and the gap of clearance time was within 2 weeks in most cases. The average time to achieve DNA clearance was 2.9 weeks for whole blood and 2.9 weeks for plasma. All the infants achieved clearance of CMV DNAemia both in whole blood and plasma at the end of the 6-month therapy.

### Comparison of the results of CMV viral load between the two different assay systems

**Fig. 4:**
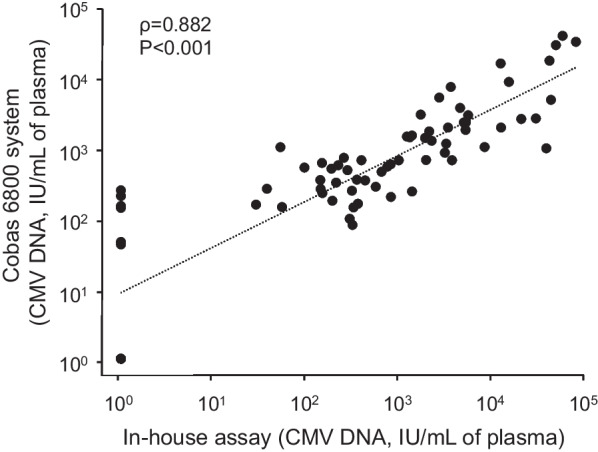
Comparison of CMV viral loads in plasma determined by the in-house real-time PCR assay and the Cobas 6800 system in patients with positive CMV results. The patients consisted of patients with cCMV (n = 20), HSCT recipients (n = 30), and liver transplantation recipients (n = 25). The viral load was compared between the two assay systems (in-house assay and the Cobas 6800 system) using Spearman’s rank correlation coefficient

To compare the quantitative results between the in-house real-time PCR assay and the Cobas 6800 system for measuring CMV viral loads, 75 plasma samples were collected from patients with cCMV (n = 20), HSCT recipients (n = 30), and liver transplantation recipients (n = 25). All samples from this “plasma validation group” were positive PCR for CMV and were retrospectively analyzed using both assays. A significant correlation was observed between the results of both assays (Fig. 4).

## Discussion

Ganciclovir and valganciclovir are approved for induction and maintenance treatment of retinitis caused by CMV infection in immunocompromised adult patients [18]. These drugs are used to treat CMV infections of other sites, such as the colon and lungs, and for the preemptive treatment of immunosuppressed adults with CMV viremia. For these cases, both drugs are administered in the early phase of the disease and are discontinued depending on clinical improvement and the clearance of viremia. In contrast, oral valganciclovir for cCMV treatment continues for up to 6 months even when the clearance of viremia is achieved because the purpose is to improve audiological and neurological outcomes.

During VGCV therapy for cCMV, measurement of CMV viral loads is preferred to evaluate the effects of VGCV if it is available. Whole blood samples have generally been used in previous studies for VGCV therapy [7, 19], but plasma samples have been widely used to assess CMV viral loads in CMV-associated diseases. In addition, commercially standardized assay systems for CMV using plasma samples have been developed [17]. Therefore, an evaluation of the usefulness of plasma samples during VGCV studies is needed to spread VGCV therapy. This is the first study to evaluate the kinetics of plasma CMV viral loads in patients with cCMV under VGCV therapy. In this study, a significant correlation between CMV levels in whole blood and plasma was observed. The levels of CMV in the whole blood were generally 10 times higher than those in the plasma. These results were consistent with previous studies on HSCT and organ transplantation [12–14].

Two types of inconsistent qualitative result patterns (whole blood+ /plasma− pattern or whole blood−/plasma+ pattern) were observed in some of the samples. An explanation for the whole blood+ /plasma− pattern is that CMV infection is mainly cell-associated [20, 21], and the cell compartment of whole blood includes relatively higher viral DNA than the plasma compartment. It is also possible to cause a whole blood−/plasma+ pattern. In the plasma samples showing this pattern, the levels of CMV were relatively low within the dynamic range of the assay (under approximately 250 IU/mL). A higher sensitivity of plasma samples has been reported in previous studies [12, 20]. This may be explained by the sample preparation step; whole blood samples are more influenced by the lysis step for the cell compartment during DNA extraction in the plasma. These results demonstrate that plasma samples are useful for monitoring CMV viral loads during VGCV therapy in whole blood samples. Finally, plasma has several advantages for use in assays. Plasma can be frozen for storage before the assay; therefore, its transportation to a commercial laboratory is feasible. In contrast, whole blood samples are not appropriate for freezing before DNA extraction because DNA yields are adversely affected [22].

The 2 cases with a high CMV level required a longer time to achieve under-detection of CMV DNA. These findings were observed in both the whole blood and plasma samples. A higher CMV level before therapy in whole blood implies worse hearing outcomes in several clinical studies [23–26]. In addition, a previous study demonstrated that lower viral loads, computed by the area under the curve of the viral loads, were correlated with better hearing outcomes during VGCV therapy [4]. Therefore, monitoring CMV levels in the peripheral blood, either whole blood or plasma, should be applied to predict hearing outcomes in patients with cCMV.

The results from quantitative PCR assays occasionally show discordant amounts of viral DNA/RNA among the different assay systems. This study compared the quantitative results of the in-house real-time PCR assay and Cobas 6800 system. As a result, there was clear concordance between the in-house assay and the Cobas 6800 assay, while variability among different PCR assay systems was reported [27, 28]. This is because both assay systems were calibrated using the 1st WHO international unit standard for human CMV for nucleic acid amplification technology [12, 29].

In this study, the kinetics of CMV viral loads in patients with cCMV were measured using whole blood, and the plasma was separated from the same whole blood. The CMV viral loads from the in-house assay were compared with those in a commercially available system, enable the plasma viral loads can be early introduced in the treatment for cCMV. However, the number of samples that were negative for both whole blood and plasma was not small. Additionally, the plasma samples in the clinical trials were not available for comparison between the in-house realtime PCR assay and Cobas 6800 system.

In conclusion, a significant correlation was observed between CMV DNA levels in whole blood and plasma samples. Higher CMV levels in the whole blood or plasma may predict prolonged viremia during VGCV therapy. The standardized commercial quantitative PCR assay for CMV using plasma can be used to monitor CMV viral loads in patients undergoing therapy.

## Data Availability

The datasets analyzed during the current study are available from the corresponding author, Y. I., on reasonable request.
